# Haplotype-based score test for linkage in nuclear families

**DOI:** 10.1186/1471-2105-8-277

**Published:** 2007-08-01

**Authors:** Chung Mo Nam, Dae Ryong Kang, Jinheum Kim

**Affiliations:** 1 Department of Preventive Medicine and Public Health, Yonsei University College of Medicine, Seoul, South Korea; 2 Department of Applied Statistics, University of Suwon, Suwon, South Korea

## Abstract

**Background:**

To look for genetic linkage between angiotensin-I converting enzyme(ACE) gene and hypertension in a Korean adolescent cohort, we developed a powerful test using the covariances between marginal differences and their variances in a transmission/non-transmission table.

**Results:**

We estimated haplotype frequencies using the parental and affected offspring's genotypes and then constructed a transmission/non-transmission table for the parental haplotypes transmitted to the offspring. We then proposed a test for checking the marginal homogeneity in the table. Because the cells in the table were dependent due to the uncertainty of the parental haplotypes, we adopted a randomization procedure to estimate the significance of the observed test statistic. Simulations show that our test performs well on a nominal level and has a monotone power, which increases as the relative risk increases. With our test, there was no evidence of genetic linkage between the ACE gene and hypertension in the Korean adolescent cohort.

**Conclusion:**

We developed a score test for linkage and used simulations to demonstrate that our test performs well at a nominal level. Under some situations where the diversity of haplotypes is low, the proposed test gained a little power over the method based on only variances between marginal differences in a transmission/non-transmission table.

## Background

For linkage and/or association studies based on haplotypes, molecular haplotyping can be done for each individual, but at a high cost. Instead, statistical methods such as Clark's algorithm [1], the EM algorithm [2], or Gibb's sampler method [3], are commonly used to reconstruct haplotypes. The likelihood ratio test, which is based on the difference between the sum of the log-likelihoods of the case and control groups and the log-likelihood of the combined data, is usually used for case-control association studies [4], while the TDT is used for linkage and/or association studies in nuclear families. The latter compares the frequency of transmitted parental haplotypes with that of non-transmitted parental haplotypes to an affected offspring. One of the difficulties with the TDT is that there is uncertainty in estimating haplotype frequencies from parental genotypes. Wilson [5] and Clayton and Jones [6] proposed that these uncertain families be discarded from the analysis, but this drop in families leads to a low power. Clayton [7] proposed an estimating procedure based on likelihood, but it is no longer robust for population admixture or population stratification. Zhao et al [8] extended Spielman and Ewens' method [9] to test for marginal homogeneity in the transmission and non-transmission of haplotypes. Their method is advantageous over those of Wilson [5] and Clayton and Jones [6] because there is no discarding of unresolved families, and it is still robust for cases of population admixture or population stratification. Cordell and Clayton [10] modeled the nuclear-family data via a conditional logistic regression in which they based on either haplotypes if parental phase may be inferrable or genotypes otherwise. Horvath et al [11] proposed family-based association tests with tightly linked markers where the phase may be ambiguous and the parental genotype data is missing. Their weighted conditional approach extended the method provided by Rabinowitz and Laird [12] to multiple markers.

Here, assuming that the haplotype block is very tight so that there is no recombination, we propose a score test for linkage and investigate its performance through simulations. We also illustrate the use of the proposed test with a data set taken from the Kangwha study [13].

## Methods and Results

### Score test

Let {*H*_*s*_*H*_*u*_, *H*_*t *_*H*_*v*_} denote the event in which the transmitted haplotype in the father is *H*_*s *_and the non-transmitted haplotype is *H*_*t*_, and simultaneously the transmitted haplotype in the mother is *H*_*u *_and the non-transmitted haplotype is *H*_*v*_. We designate {*H*_*s*_*H*_*u*_, *H*_*t*_*H*_*v*_} as one haplotype group and define {*H*_*s^g^*_*H*_*u^g^*_, *H*_*t^g^*_*H*_*v^g^*_} as haplotype groups corresponding to the set of genotypes *g *= 1, ..., *G*, where *G *is the number of distinct sets of genotypes across all markers. Here each set of genotypes refers to the observed genotypes for the individual markers of the two parents and the affected offspring. Suppose the total number of possible haplotypes is *k*.

Zhao et al [8] proposed a TDT statistic to test linkage between markers and disease genes. They obtained a set of the estimated haplotype frequencies, {h˜i
 MathType@MTEF@5@5@+=feaafiart1ev1aaatCvAUfKttLearuWrP9MDH5MBPbIqV92AaeXatLxBI9gBaebbnrfifHhDYfgasaacH8akY=wiFfYdH8Gipec8Eeeu0xXdbba9frFj0=OqFfea0dXdd9vqai=hGuQ8kuc9pgc9s8qqaq=dirpe0xb9q8qiLsFr0=vr0=vr0dc8meaabaqaciaacaGaaeqabaqabeGadaaakeaacuWGObaAgaacamaaBaaaleaacqWGPbqAaeqaaaaa@2F9B@}, using the EM algorithm based on the parental genotypes only, and for the haplotype group {*H*_*s*_*H*_*u*_, *H*_*t*_*H*_*v*_} compatible with the set of genotypes *g*, defined t˜gsu,tv
 MathType@MTEF@5@5@+=feaafiart1ev1aaatCvAUfKttLearuWrP9MDH5MBPbIqV92AaeXatLxBI9gBaebbnrfifHhDYfgasaacH8akY=wiFfYdH8Gipec8Eeeu0xXdbba9frFj0=OqFfea0dXdd9vqai=hGuQ8kuc9pgc9s8qqaq=dirpe0xb9q8qiLsFr0=vr0=vr0dc8meaabaqaciaacaGaaeqabaqabeGadaaakeaacuWG0baDgaacamaaDaaaleaacqWGNbWzaeaacqWGZbWCcqWG1bqDcqGGSaalcqWG0baDcqWG2bGDaaaaaa@3658@ as the estimated number of families in which the father with haplotypes {*H*_*s*_, *H*_*t*_} transmits *H*_*s *_and the mother with haplotypes {*H*_*u*_, *H*_*v*_} transmits *H*_*u*_. Here the value of t˜gsu,tv
 MathType@MTEF@5@5@+=feaafiart1ev1aaatCvAUfKttLearuWrP9MDH5MBPbIqV92AaeXatLxBI9gBaebbnrfifHhDYfgasaacH8akY=wiFfYdH8Gipec8Eeeu0xXdbba9frFj0=OqFfea0dXdd9vqai=hGuQ8kuc9pgc9s8qqaq=dirpe0xb9q8qiLsFr0=vr0=vr0dc8meaabaqaciaacaGaaeqabaqabeGadaaakeaacuWG0baDgaacamaaDaaaleaacqWGNbWzaeaacqWGZbWCcqWG1bqDcqGGSaalcqWG0baDcqWG2bGDaaaaaa@3658@ is given by

t˜gsu,tv=ngh˜sh˜th˜uh˜v∑h˜sgh˜tgh˜ugh˜vg,
 MathType@MTEF@5@5@+=feaafiart1ev1aaatCvAUfKttLearuWrP9MDH5MBPbIqV92AaeXatLxBI9gBaebbnrfifHhDYfgasaacH8akY=wiFfYdH8Gipec8Eeeu0xXdbba9frFj0=OqFfea0dXdd9vqai=hGuQ8kuc9pgc9s8qqaq=dirpe0xb9q8qiLsFr0=vr0=vr0dc8meaabaqaciaacaGaaeqabaqabeGadaaakeaacuWG0baDgaacamaaDaaaleaacqWGNbWzaeaacqWGZbWCcqWG1bqDcqGGSaalcqWG0baDcqWG2bGDaaGccqGH9aqpcqWGUbGBdaWgaaWcbaGaem4zaCgabeaakmaalaaabaGafmiAaGMbaGaadaWgaaWcbaGaem4CamhabeaakiqbdIgaOzaaiaWaaSbaaSqaaiabdsha0bqabaGccuWGObaAgaacamaaBaaaleaacqWG1bqDaeqaaOGafmiAaGMbaGaadaWgaaWcbaGaemODayhabeaaaOqaamaaqaeabaGafmiAaGMbaGaadaWgaaWcbaGaem4Cam3aaWbaaWqabeaacqWGNbWzaaaaleqaaOGafmiAaGMbaGaadaWgaaWcbaGaemiDaq3aaWbaaWqabeaacqWGNbWzaaaaleqaaOGafmiAaGMbaGaadaWgaaWcbaGaemyDau3aaWbaaWqabeaacqWGNbWzaaaaleqaaOGafmiAaGMbaGaadaWgaaWcbaGaemODay3aaWbaaWqabeaacqWGNbWzaaaaleqaaaqabeqaniabggHiLdaaaOGaeiilaWcaaa@5C13@

where the summation is over all haplotype groups compatible with *g*, and *n*_*g *_is the total number of families compatible with *g*. Then they constructed a *k *× *k *transmission/non-transmission table T˜
 MathType@MTEF@5@5@+=feaafiart1ev1aaatCvAUfKttLearuWrP9MDH5MBPbIqV92AaeXatLxBI9gBaebbnrfifHhDYfgasaacH8akY=wiFfYdH8Gipec8Eeeu0xXdbba9frFj0=OqFfea0dXdd9vqai=hGuQ8kuc9pgc9s8qqaq=dirpe0xb9q8qiLsFr0=vr0=vr0dc8meaabaqaciaacaGaaeqabaqabeGadaaakeaaieqacuWFubavgaacaaaa@2DF2@ = {t˜ij
 MathType@MTEF@5@5@+=feaafiart1ev1aaatCvAUfKttLearuWrP9MDH5MBPbIqV92AaeXatLxBI9gBaebbnrfifHhDYfgasaacH8akY=wiFfYdH8Gipec8Eeeu0xXdbba9frFj0=OqFfea0dXdd9vqai=hGuQ8kuc9pgc9s8qqaq=dirpe0xb9q8qiLsFr0=vr0=vr0dc8meaabaqaciaacaGaaeqabaqabeGadaaakeaacuWG0baDgaacamaaBaaaleaacqWGPbqAcqWGQbGAaeqaaaaa@3110@}, where t˜ij
 MathType@MTEF@5@5@+=feaafiart1ev1aaatCvAUfKttLearuWrP9MDH5MBPbIqV92AaeXatLxBI9gBaebbnrfifHhDYfgasaacH8akY=wiFfYdH8Gipec8Eeeu0xXdbba9frFj0=OqFfea0dXdd9vqai=hGuQ8kuc9pgc9s8qqaq=dirpe0xb9q8qiLsFr0=vr0=vr0dc8meaabaqaciaacaGaaeqabaqabeGadaaakeaacuWG0baDgaacamaaBaaaleaacqWGPbqAcqWGQbGAaeqaaaaa@3110@ is the estimated number of parents who have haplotypes {*H*_*i*_, *H*_*j*_} and who transmit *H*_*i *_to the affected offspring across all sets of genotypes. That is,

t˜ij=∑g{∑u∑vt˜giu,jv+∑s∑tt˜gsi,tj},
 MathType@MTEF@5@5@+=feaafiart1ev1aaatCvAUfKttLearuWrP9MDH5MBPbIqV92AaeXatLxBI9gBaebbnrfifHhDYfgasaacH8akY=wiFfYdH8Gipec8Eeeu0xXdbba9frFj0=OqFfea0dXdd9vqai=hGuQ8kuc9pgc9s8qqaq=dirpe0xb9q8qiLsFr0=vr0=vr0dc8meaabaqaciaacaGaaeqabaqabeGadaaakeaacuWG0baDgaacamaaBaaaleaacqWGPbqAcqWGQbGAaeqaaOGaeyypa0ZaaabuaeaadaGadeqaamaaqafabaWaaabuaeaacuWG0baDgaacamaaDaaaleaacqWGNbWzaeaacqWGPbqAcqWG1bqDcqGGSaalcqWGQbGAcqWG2bGDaaaabaGaemODayhabeqdcqGHris5aOGaey4kaSYaaabuaeaadaaeqbqaaiqbdsha0zaaiaWaa0baaSqaaiabdEgaNbqaaiabdohaZjabdMgaPjabcYcaSiabdsha0jabdQgaQbaaaeaacqWG0baDaeqaniabggHiLdaaleaacqWGZbWCaeqaniabggHiLdaaleaacqWG1bqDaeqaniabggHiLdaakiaawUhacaGL9baaaSqaaiabdEgaNbqab0GaeyyeIuoakiabcYcaSaaa@5AEA@

where the summation over *g *ranges from 1 to *G *and the summations over *s*, *t*, *u*, and *v *range from 1 to *k*. They derived a test applying Spielman and Ewens' method to the table T˜
 MathType@MTEF@5@5@+=feaafiart1ev1aaatCvAUfKttLearuWrP9MDH5MBPbIqV92AaeXatLxBI9gBaebbnrfifHhDYfgasaacH8akY=wiFfYdH8Gipec8Eeeu0xXdbba9frFj0=OqFfea0dXdd9vqai=hGuQ8kuc9pgc9s8qqaq=dirpe0xb9q8qiLsFr0=vr0=vr0dc8meaabaqaciaacaGaaeqabaqabeGadaaakeaaieqacuWFubavgaacaaaa@2DF2@.

In this work we propose a score test that extends Sham's method [14] by modifying the transmission/non-transmission table T˜
 MathType@MTEF@5@5@+=feaafiart1ev1aaatCvAUfKttLearuWrP9MDH5MBPbIqV92AaeXatLxBI9gBaebbnrfifHhDYfgasaacH8akY=wiFfYdH8Gipec8Eeeu0xXdbba9frFj0=OqFfea0dXdd9vqai=hGuQ8kuc9pgc9s8qqaq=dirpe0xb9q8qiLsFr0=vr0=vr0dc8meaabaqaciaacaGaaeqabaqabeGadaaakeaaieqacuWFubavgaacaaaa@2DF2@. As mentioned by Rohde and Fuerst [15], the haplotype frequency estimation done using the EM algorithm over independent parents may derive possible haplotype pairs for parents that are contradictory to the ones for the offspring. However, including the offspring's genotype in the haplotype frequency estimation makes it possible to exclude these misleading haplotype pairs, thereby improving the accuracy of the underlying haplotype pair and the accuracy of a transmission/non-transmission table. This is why we adopted the EM method proposed by Rohde and Fuerst [15] and Becker and Knapp [16] rather than the method proposed by Long, Williams, and Urbanek [2]. We first obtained estimated haplotype frequencies, {h^i
 MathType@MTEF@5@5@+=feaafiart1ev1aaatCvAUfKttLearuWrP9MDH5MBPbIqV92AaeXatLxBI9gBaebbnrfifHhDYfgasaacH8akY=wiFfYdH8Gipec8Eeeu0xXdbba9frFj0=OqFfea0dXdd9vqai=hGuQ8kuc9pgc9s8qqaq=dirpe0xb9q8qiLsFr0=vr0=vr0dc8meaabaqaciaacaGaaeqabaqabeGadaaakeaacuWGObaAgaqcamaaBaaaleaacqWGPbqAaeqaaaaa@2F9C@}, based on family trios instead of on independent parental genotypes. Replacing h˜i
 MathType@MTEF@5@5@+=feaafiart1ev1aaatCvAUfKttLearuWrP9MDH5MBPbIqV92AaeXatLxBI9gBaebbnrfifHhDYfgasaacH8akY=wiFfYdH8Gipec8Eeeu0xXdbba9frFj0=OqFfea0dXdd9vqai=hGuQ8kuc9pgc9s8qqaq=dirpe0xb9q8qiLsFr0=vr0=vr0dc8meaabaqaciaacaGaaeqabaqabeGadaaakeaacuWGObaAgaacamaaBaaaleaacqWGPbqAaeqaaaaa@2F9B@, *i *= 1, ..., *k*, by h^i
 MathType@MTEF@5@5@+=feaafiart1ev1aaatCvAUfKttLearuWrP9MDH5MBPbIqV92AaeXatLxBI9gBaebbnrfifHhDYfgasaacH8akY=wiFfYdH8Gipec8Eeeu0xXdbba9frFj0=OqFfea0dXdd9vqai=hGuQ8kuc9pgc9s8qqaq=dirpe0xb9q8qiLsFr0=vr0=vr0dc8meaabaqaciaacaGaaeqabaqabeGadaaakeaacuWGObaAgaqcamaaBaaaleaacqWGPbqAaeqaaaaa@2F9C@ in equations (1) and (2), we denote t˜gsu,tv
 MathType@MTEF@5@5@+=feaafiart1ev1aaatCvAUfKttLearuWrP9MDH5MBPbIqV92AaeXatLxBI9gBaebbnrfifHhDYfgasaacH8akY=wiFfYdH8Gipec8Eeeu0xXdbba9frFj0=OqFfea0dXdd9vqai=hGuQ8kuc9pgc9s8qqaq=dirpe0xb9q8qiLsFr0=vr0=vr0dc8meaabaqaciaacaGaaeqabaqabeGadaaakeaacuWG0baDgaacamaaDaaaleaacqWGNbWzaeaacqWGZbWCcqWG1bqDcqGGSaalcqWG0baDcqWG2bGDaaaaaa@3658@, t˜ij
 MathType@MTEF@5@5@+=feaafiart1ev1aaatCvAUfKttLearuWrP9MDH5MBPbIqV92AaeXatLxBI9gBaebbnrfifHhDYfgasaacH8akY=wiFfYdH8Gipec8Eeeu0xXdbba9frFj0=OqFfea0dXdd9vqai=hGuQ8kuc9pgc9s8qqaq=dirpe0xb9q8qiLsFr0=vr0=vr0dc8meaabaqaciaacaGaaeqabaqabeGadaaakeaacuWG0baDgaacamaaBaaaleaacqWGPbqAcqWGQbGAaeqaaaaa@3110@, and T˜
 MathType@MTEF@5@5@+=feaafiart1ev1aaatCvAUfKttLearuWrP9MDH5MBPbIqV92AaeXatLxBI9gBaebbnrfifHhDYfgasaacH8akY=wiFfYdH8Gipec8Eeeu0xXdbba9frFj0=OqFfea0dXdd9vqai=hGuQ8kuc9pgc9s8qqaq=dirpe0xb9q8qiLsFr0=vr0=vr0dc8meaabaqaciaacaGaaeqabaqabeGadaaakeaaieqacuWFubavgaacaaaa@2DF2@ by t^gsu,tv
 MathType@MTEF@5@5@+=feaafiart1ev1aaatCvAUfKttLearuWrP9MDH5MBPbIqV92AaeXatLxBI9gBaebbnrfifHhDYfgasaacH8akY=wiFfYdH8Gipec8Eeeu0xXdbba9frFj0=OqFfea0dXdd9vqai=hGuQ8kuc9pgc9s8qqaq=dirpe0xb9q8qiLsFr0=vr0=vr0dc8meaabaqaciaacaGaaeqabaqabeGadaaakeaacuWG0baDgaqcamaaDaaaleaacqWGNbWzaeaacqWGZbWCcqWG1bqDcqGGSaalcqWG0baDcqWG2bGDaaaaaa@3659@, and t^ij
 MathType@MTEF@5@5@+=feaafiart1ev1aaatCvAUfKttLearuWrP9MDH5MBPbIqV92AaeXatLxBI9gBaebbnrfifHhDYfgasaacH8akY=wiFfYdH8Gipec8Eeeu0xXdbba9frFj0=OqFfea0dXdd9vqai=hGuQ8kuc9pgc9s8qqaq=dirpe0xb9q8qiLsFr0=vr0=vr0dc8meaabaqaciaacaGaaeqabaqabeGadaaakeaacuWG0baDgaqcamaaBaaaleaacqWGPbqAcqWGQbGAaeqaaaaa@3111@, and T^
 MathType@MTEF@5@5@+=feaafiart1ev1aaatCvAUfKttLearuWrP9MDH5MBPbIqV92AaeXatLxBI9gBamXvP5wqSXMqHnxAJn0BKvguHDwzZbqegyvzYrwyUfgarqqtubsr4rNCHbGeaGqiVu0Je9sqqrpepC0xbbL8F4rqqrFfpeea0xe9Lq=Jc9vqaqpepm0xbba9pwe9Q8fs0=yqaqpepae9pg0FirpepeKkFr0xfr=xfr=xb9adbaqaaeGacaGaaiaabeqaamqadiabaaGcbaGafmivaqLbaKaaaaa@37B9@, respectively. We let t^i.
 MathType@MTEF@5@5@+=feaafiart1ev1aaatCvAUfKttLearuWrP9MDH5MBPbIqV92AaeXatLxBI9gBaebbnrfifHhDYfgasaacH8akY=wiFfYdH8Gipec8Eeeu0xXdbba9frFj0=OqFfea0dXdd9vqai=hGuQ8kuc9pgc9s8qqaq=dirpe0xb9q8qiLsFr0=vr0=vr0dc8meaabaqaciaacaGaaeqabaqabeGadaaakeaacuWG0baDgaqcamaaBaaaleaacqWGPbqAaeqaaOGaeiOla4caaa@30A2@, *i *= 1, ..., *k*, be the row marginal totals, defined by t^i.=∑j=1kt^ij
 MathType@MTEF@5@5@+=feaafiart1ev1aaatCvAUfKttLearuWrP9MDH5MBPbIqV92AaeXatLxBI9gBaebbnrfifHhDYfgasaacH8akY=wiFfYdH8Gipec8Eeeu0xXdbba9frFj0=OqFfea0dXdd9vqai=hGuQ8kuc9pgc9s8qqaq=dirpe0xb9q8qiLsFr0=vr0=vr0dc8meaabaqaciaacaGaaeqabaqabeGadaaakeaacuWG0baDgaqcamaaBaaaleaacqWGPbqAaeqaaOGaeiOla4Iaeyypa0ZaaabmaeaacuWG0baDgaqcamaaBaaaleaacqWGPbqAcqWGQbGAaeqaaaqaaiabdQgaQjabg2da9iabigdaXaqaaiabdUgaRbqdcqGHris5aaaa@3CB6@ and t^.j
 MathType@MTEF@5@5@+=feaafiart1ev1aaatCvAUfKttLearuWrP9MDH5MBPbIqV92AaeXatLxBI9gBaebbnrfifHhDYfgasaacH8akY=wiFfYdH8Gipec8Eeeu0xXdbba9frFj0=OqFfea0dXdd9vqai=hGuQ8kuc9pgc9s8qqaq=dirpe0xb9q8qiLsFr0=vr0=vr0dc8meaabaqaciaacaGaaeqabaqabeGadaaakeaacuWG0baDgaqcaiabc6caUmaaBaaaleaacqWGQbGAaeqaaaaa@309A@, *j *= 1, ..., *k*, the column marginal totals, defined by t^.j=∑i=1kt^ij
 MathType@MTEF@5@5@+=feaafiart1ev1aaatCvAUfKttLearuWrP9MDH5MBPbIqV92AaeXatLxBI9gBaebbnrfifHhDYfgasaacH8akY=wiFfYdH8Gipec8Eeeu0xXdbba9frFj0=OqFfea0dXdd9vqai=hGuQ8kuc9pgc9s8qqaq=dirpe0xb9q8qiLsFr0=vr0=vr0dc8meaabaqaciaacaGaaeqabaqabeGadaaakeaacuWG0baDgaqcaiabc6caUmaaBaaaleaacqWGQbGAaeqaaOGaeyypa0ZaaabmaeaacuWG0baDgaqcamaaBaaaleaacqWGPbqAcqWGQbGAaeqaaaqaaiabdMgaPjabg2da9iabigdaXaqaaiabdUgaRbqdcqGHris5aaaa@3CB6@. We denote a vector of the marginal discrepancies for haplotypes 1 to *k *- 1 by Δ^=(t^1.−t^.1,...,t^k−1.−t^.k−1)′
 MathType@MTEF@5@5@+=feaafiart1ev1aaatCvAUfKttLearuWrP9MDH5MBPbIqV92AaeXatLxBI9gBaebbnrfifHhDYfgasaacH8akY=wiFfYdH8Gipec8Eeeu0xXdbba9frFj0=OqFfea0dXdd9vqai=hGuQ8kuc9pgc9s8qqaq=dirpe0xb9q8qiLsFr0=vr0=vr0dc8meaabaqaciaacaGaaeqabaqabeGadaaakeaaiiqacuWFuoargaqcaiabg2da9iabcIcaOiqbdsha0zaajaWaaSbaaSqaaiabigdaXaqabaGccqGGUaGlcqGHsislcuWG0baDgaqcaiabc6caUmaaBaaaleaacqaIXaqmaeqaaOGaeiilaWIaeiOla4IaeiOla4IaeiOla4IaeiilaWIafmiDaqNbaKaadaWgaaWcbaGaem4AaSMaeyOeI0IaeGymaedabeaakiabc6caUiabgkHiTiqbdsha0zaajaGaeiOla4YaaSbaaSqaaiabdUgaRjabgkHiTiabigdaXaqabaGccuGGPaqkgaqbaaaa@49F5@. Note that when there is no linkage, *E*(Δ^
 MathType@MTEF@5@5@+=feaafiart1ev1aaatCvAUfKttLearuWrP9MDH5MBPbIqV92AaeXatLxBI9gBaebbnrfifHhDYfgasaacH8akY=wiFfYdH8Gipec8Eeeu0xXdbba9frFj0=OqFfea0dXdd9vqai=hGuQ8kuc9pgc9s8qqaq=dirpe0xb9q8qiLsFr0=vr0=vr0dc8meaabaqaciaacaGaaeqabaqabeGadaaakeaaiiqacuWFuoargaqcaaaa@2E27@) = (0, ..., 0)*' *because the matrix t^i,j
 MathType@MTEF@5@5@+=feaafiart1ev1aaatCvAUfKttLearuWrP9MDH5MBPbIqV92AaeXatLxBI9gBaebbnrfifHhDYfgasaacH8akY=wiFfYdH8Gipec8Eeeu0xXdbba9frFj0=OqFfea0dXdd9vqai=hGuQ8kuc9pgc9s8qqaq=dirpe0xb9q8qiLsFr0=vr0=vr0dc8meaabaqaciaacaGaaeqabaqabeGadaaakeaacuWG0baDgaqcamaaBaaaleaacqWGPbqAcqGGSaalcqWGQbGAaeqaaaaa@31F1@ is symmetrical under the null hypothesis of no linkage irrespective of a set of haplotype frequencies [8]. Letting Σ^
 MathType@MTEF@5@5@+=feaafiart1ev1aaatCvAUfKttLearuWrP9MDH5MBPbIqV92AaeXatLxBI9gBaebbnrfifHhDYfgasaacH8akY=wiFfYdH8Gipec8Eeeu0xXdbba9frFj0=OqFfea0dXdd9vqai=hGuQ8kuc9pgc9s8qqaq=dirpe0xb9q8qiLsFr0=vr0=vr0dc8meaabaqaciaacaGaaeqabaqabeGadaaakeaaiiqacuWFJoWugaqcaaaa@2E45@ = {σ^ij
 MathType@MTEF@5@5@+=feaafiart1ev1aaatCvAUfKttLearuWrP9MDH5MBPbIqV92AaeXatLxBI9gBaebbnrfifHhDYfgasaacH8akY=wiFfYdH8Gipec8Eeeu0xXdbba9frFj0=OqFfea0dXdd9vqai=hGuQ8kuc9pgc9s8qqaq=dirpe0xb9q8qiLsFr0=vr0=vr0dc8meaabaqaciaacaGaaeqabaqabeGadaaakeaaiiGacuWFdpWCgaqcamaaBaaaleaacqWGPbqAcqWGQbGAaeqaaaaa@316A@} with diagonal elements σ^ii=t^i.+t^.i−2t^ii
 MathType@MTEF@5@5@+=feaafiart1ev1aaatCvAUfKttLearuWrP9MDH5MBPbIqV92AaeXatLxBI9gBaebbnrfifHhDYfgasaacH8akY=wiFfYdH8Gipec8Eeeu0xXdbba9frFj0=OqFfea0dXdd9vqai=hGuQ8kuc9pgc9s8qqaq=dirpe0xb9q8qiLsFr0=vr0=vr0dc8meaabaqaciaacaGaaeqabaqabeGadaaakeaaiiGacuWFdpWCgaqcamaaBaaaleaacqWGPbqAcqWGPbqAaeqaaOGaeyypa0JafmiDaqNbaKaadaWgaaWcbaGaemyAaKgabeaakiabc6caUiabgUcaRiqbdsha0zaajaGaeiOla4YaaSbaaSqaaiabdMgaPbqabaGccqGHsislcqaIYaGmcuWG0baDgaqcamaaBaaaleaacqWGPbqAcqWGPbqAaeqaaaaa@4188@ and off-diagonal elements σ^ij=−(t^ij+t^ji);(i≠j)
 MathType@MTEF@5@5@+=feaafiart1ev1aaatCvAUfKttLearuWrP9MDH5MBPbIqV92AaeXatLxBI9gBaebbnrfifHhDYfgasaacH8akY=wiFfYdH8Gipec8Eeeu0xXdbba9frFj0=OqFfea0dXdd9vqai=hGuQ8kuc9pgc9s8qqaq=dirpe0xb9q8qiLsFr0=vr0=vr0dc8meaabaqaciaacaGaaeqabaqabeGadaaakeaaiiGacuWFdpWCgaqcamaaBaaaleaacqWGPbqAcqWGQbGAaeqaaOGaeyypa0JaeyOeI0IaeiikaGIafmiDaqNbaKaadaWgaaWcbaGaemyAaKMaemOAaOgabeaakiabgUcaRiqbdsha0zaajaWaaSbaaSqaaiabdQgaQjabdMgaPbqabaGccqGGPaqkcqGG7aWocqGGOaakcqWGPbqAcqGHGjsUcqWGQbGAcqGGPaqkaaa@4608@, we propose a score test statistic *T*_*s *_defined by

Ts=Δ^′Σ^−1Δ^.
 MathType@MTEF@5@5@+=feaafiart1ev1aaatCvAUfKttLearuWrP9MDH5MBPbIqV92AaeXatLxBI9gBaebbnrfifHhDYfgasaacH8akY=wiFfYdH8Gipec8Eeeu0xXdbba9frFj0=OqFfea0dXdd9vqai=hGuQ8kuc9pgc9s8qqaq=dirpe0xb9q8qiLsFr0=vr0=vr0dc8meaabaqaciaacaGaaeqabaqabeGadaaakeaacqWGubavdaWgaaWcbaGaem4Camhabeaakiabg2da9GGabiqb=r5aezaajyaafaGaf83OdmLbaKaadaahaaWcbeqaaiabgkHiTiabigdaXaaakiqb=r5aezaajaGaeiOla4caaa@3804@

Whenever all the parental haplotype phases are uniquely determined, *T*_*s *_asymptotically follows a chi-square distribution with (*k *- 1) degrees of freedom because Σ^
 MathType@MTEF@5@5@+=feaafiart1ev1aaatCvAUfKttLearuWrP9MDH5MBPbIqV92AaeXatLxBI9gBaebbnrfifHhDYfgasaacH8akY=wiFfYdH8Gipec8Eeeu0xXdbba9frFj0=OqFfea0dXdd9vqai=hGuQ8kuc9pgc9s8qqaq=dirpe0xb9q8qiLsFr0=vr0=vr0dc8meaabaqaciaacaGaaeqabaqabeGadaaakeaaiiqacuWFJoWugaqcaaaa@2E45@ is the covariance matrix of Δ^
 MathType@MTEF@5@5@+=feaafiart1ev1aaatCvAUfKttLearuWrP9MDH5MBPbIqV92AaeXatLxBI9gBaebbnrfifHhDYfgasaacH8akY=wiFfYdH8Gipec8Eeeu0xXdbba9frFj0=OqFfea0dXdd9vqai=hGuQ8kuc9pgc9s8qqaq=dirpe0xb9q8qiLsFr0=vr0=vr0dc8meaabaqaciaacaGaaeqabaqabeGadaaakeaaiiqacuWFuoargaqcaaaa@2E27@ under no linkage (see Appendix for the details). We note, however, that the cells in T^
 MathType@MTEF@5@5@+=feaafiart1ev1aaatCvAUfKttLearuWrP9MDH5MBPbIqV92AaeXatLxBI9gBamXvP5wqSXMqHnxAJn0BKvguHDwzZbqegyvzYrwyUfgarqqtubsr4rNCHbGeaGqiVu0Je9sqqrpepC0xbbL8F4rqqrFfpeea0xe9Lq=Jc9vqaqpepm0xbba9pwe9Q8fs0=yqaqpepae9pg0FirpepeKkFr0xfr=xfr=xb9adbaqaaeGacaGaaiaabeqaamqadiabaaGcbaGafmivaqLbaKaaaaa@37B9@ are not independent because the contribution of a father or a mother is not limited to a cell because of its uncertainty as a haplotype pair, but reaches out to some cells according to the number of possible haplotype pairs and corresponding probabilities. That is why *T*_*s *_may not have a chi-square distribution with (*k *- 1) degrees of freedom. Thus, we adopted the randomization test procedure introduced by Zhao et al [8] to estimate the statistical significance of the proposed test instead of relying on an asymptotic chi-square test.

### Simulation studies

In our simulations, a hypothetical genealogy with three biallelic genetic markers was considered. We denoted alleles at the unobservable disease-susceptibility locus as *D *and *d*. There are eight haplotypes consisting of bialleles at three loci, *l*_1_, *l*_2_, and *l*_3_: *H*_1 _= (1,1,1), *H*_2 _= (1,1,2), *H*_3 _= (1,2,1), *H*_4 _= (1,2,2), *H*_5 _= (2,1,1), *H*_6 _= (2,1,2), *H*_7 _= (2,2,1), and *H*_8 _= (2,2,2). Here both *H*_7 _and *H*_8 _include the disease-susceptibility allele *d*, whereas the other six haplotypes include the wild-type allele *D*. For each *i *= 1, ..., 8, let *h*_*i *_be the frequency of *H*_*i*_. We set 4 types of haplotype distribution: (*h*_1_, *h*_2_, *h*_3_, *h*_4_, *h*_5_, *h*_6_, *h*_7_, *h*_8_) = (.125, .125, .125, .125, .125, .125, .125, .125), (.343, .147, .147, .063, .147, .063, .027), (.343, .147, .210, .000, .210, .000, .063, .027), and (.700, .000, .000, .000, .210, .000, .063, .027), denoted as 'E', 'UE1', 'UE2', and 'UE3', respectively. Note that the degree of LD between each marker at loci, *l*_1 _and *l*_2_, and the disease marker is high (*D' *= 1), while the LD between the marker at a locus *l*_3 _and the marker for the disease is relatively low (*D' *= 0 for E and UE1, *D' *= 0.15 for UE2, and *D' *= 1 for UE3). The subjects with the *d *allele have experienced mutations at loci, *l*_1 _and *l*_2_, whereas the mutation at a locus *l*_3 _happened irrespective of the disease marker allele. Both dominant and recessive models were considered for the mode of inheritance. We assumed Hardy-Weinberg equilibrium and random-mating and that the families were ascertained through one affected offspring.

For the dominant genetic models, if an offspring has at least one of the high risk haplotypes, *H*_7 _and *H*_8_, its penetrance equals to *c *× RR; otherwise, the individual who does not carry the disease-susceptibility allele *d *may be affected with a probability of *c*. For the recessive genetic models, only subjects having both *H*_7 _and *H*_8_, *H*_7 _homozygote, or *H*_8 _homozygote may have the penetrance of *c *× RR, and the rest may be affected with a probability of *c*. We set *c *as 0.1 and RR as 1, 2, 3, and 4. Here RR = 1 corresponds to level and the rest to power. We ascertained 100 families and 200 independent samples were generated for each simulation model. We estimated the significance level of the tests based on 100 randomly generated samples from each sample in the study of type I error rate and power. Table 1 summarizes the estimated type I error rates and powers of the proposed and Zhao et al's tests at a significance level of 0.05. Note that the standard error of the type I error rate estimate is 0.015. Additionally, entries in columns denoted by '*p*_*f *_' and '*p*_*o*_' represent the average percentages of perfectly estimated haplotypes for 100 families, respectively, when based on family trios and based on parents alone in estimating haplotype frequencies.

**Table 1:** Empirical type I error rates and powers of the proposed (*T*_*s*_) and Zhao et al's (*T*_*z*_) tests, and average percentages of perfectly estimated haplotypes for the dominant and recessive models according to 4 types of haplotype distribution (HD)

		Dominant				Recessive			
			
HD	RR	*p*_*f*_	*p*_*o*_	*T*_*s*_	*T*_*z*_	*p*_*f*_	*p*_*o*_	*T*_*s*_	*T*_*z*_
E	1	88	72	.060	.065	88	72	.045	.050
	2	88	73	.275	.280	88	72	.145	.110
	3	88	73	.635	.645	89	73	.275	.275
	4	89	74	.815	.820	89	72	.455	.460
									
UE1	1	92	79	.060	.065	92	79	.050	.040
	2	92	79	.240	.235	92	79	.060	.050
	3	92	79	.560	.545	92	79	.050	.055
	4	92	79	.845	.840	93	79	.065	.055
									
UE2	1	99	94	.050	.065	99	94	.075	.085
	2	98	92	.270	.250	99	93	.035	.040
	3	98	90	.610	.580	99	93	.065	.055
	4	97	89	.885	.860	99	93	.065	.060
									
UE3	1	100	100	.040	.055	100	100	.090	.075
	2	100	100	.375	.325	100	100	.055	.065
	3	100	100	.795	.780	100	100	.060	.055
	4	100	100	.960	.950	100	100	.070	.060

The rows corresponding to RR = 1 are for type I error rate, and the rows corresponding to RR = 2, 3 or 4 are for power. The columns corresponding to *p*_*f *_and *p*_*o *_are the average percentages of perfectly estimated haplotypes, respectively, when based on family trios and based on parents alone in estimating haplotype frequencies.

As mentioned by Rohde and Fuerst [15], the percentage of perfectly estimated haplotypes is higher when estimates are based on family trios rather than on parents alone. Therefore, using the offspring's genotype can reduce uncertainty in parental haplotypes. As expected, the lower the diversity of haplotypes, the higher the percentage of perfect haplotype estimates. For example, the percentage of perfectly estimated haplotypes for the UE1 case was higher than for the E case, and the percentage was higher for the UE2 or UE3 case than for the UE1 case. However, there was no difference in percentages between dominant and recessive models.

Based on our results with the proposed test, shown in entries corresponding to RR = 1, the estimated type I error rate seems to satisfy a nominal level of 0.05, regardless of the mode of inheritance and type of haplotype distribution. For the dominant models, the power of the test increases as RR increases from 2 to 4, irrespective of the type of haplotype distribution. In the recessive models, the power increases as RR increases for the E case, while the power equals to the nominal level for the UE1, UE2, and UE3 cases. This explains why the probability of a proband having the mutant homozygotes is very low at 0.006 for the UE1, UE2, and UE3 cases relative to 0.047 for the E case.

We also compared the proposed test and Zhao et al's test in terms of type I error rate and power. Both tests showed comparable performance at a significance level of 0.05. For the E case, Zhao et al's test was more powerful than our test; for the UE1, UE2, and UE3 cases, however, the latter had better power than the former. It seems that as the diversity of haplotypes decreases, the off-diagonal entries of T^
 MathType@MTEF@5@5@+=feaafiart1ev1aaatCvAUfKttLearuWrP9MDH5MBPbIqV92AaeXatLxBI9gBamXvP5wqSXMqHnxAJn0BKvguHDwzZbqegyvzYrwyUfgarqqtubsr4rNCHbGeaGqiVu0Je9sqqrpepC0xbbL8F4rqqrFfpeea0xe9Lq=Jc9vqaqpepm0xbba9pwe9Q8fs0=yqaqpepae9pg0FirpepeKkFr0xfr=xfr=xb9adbaqaaeGacaGaaiaabeqaamqadiabaaGcbaGafmivaqLbaKaaaaa@37B9@ correspondingly become more unbalanced and thereby are as informative as the marginal totals and this results in our test being more powerful than Zhao et al's test.

Finally, we performed simulations to investigate the robustness of the proposed test in cases of population admixture and to determine the conservativeness of the asymptotic chi-squared test. To this end, the haplotype distribution of a new population to be mixed with E and UE1, UE2 or UE3, respectively, was taken as (*h*_1_, *h*_2_, *h*_3_, *h*_4_, *h*_5_, *h*_6_, *h*_7_, *h*_8_) = (.250, .000, .250, .000, .250, .000, .250, .000) and (.490, .000, .210, 000, .210, .000, .090, .000), denoted as 'E-3' and 'UE-3'. These new populations have no mutation at a locus *l*_3 _in common. We set *c *as 0.2 for the 'E-3' and 'UE-3' cases. We considered three types of proportion for the two populations in the sample size; 1:1, 1:3, and 3:1, denoted as 'P11', 'P13', and 'P31', respectively. The total number of ascertained families in the mixed population was 100. Table 2 presents the empirical and asymptotic type I error rates of our test along with Zhao et al's test with the dominant model at a nominal level of 0.05. Based on various situations incorporated in the simulations, we have shown that both our proposed test and Zhao et al's test are robust to population admixture. As mentioned in Section Score test, we also determined that the asymptotic chi-squared tests were conservative through simulations.

**Table 2:** Empirical and asymptotic type I error rates of the proposed (*T*_*s*_) and Zhao et al's (*T*_*z*_) tests for the dominant model according to all the combinations of 4 pairs of haplotype distributions (HDs) and 3 types of proportion for the two populations in sample size (PP)

		Empirical		Asymptotic	
			
Pair of HDs	PP	*T*_*s*_	*T*_*z*_	*T*_*s*_	*T*_*z*_
E & E-3	P11	.055	.045	.025	.020
	P13	.050	.045	.015	.030
	P31	.060	.035	.035	.040
					
UE1 & UE-3	P11	.060	.055	.025	.035
	P13	.055	.075	.020	.025
	P31	.050	.040	.030	.030
					
UE2 & UE-3	P11	.060	.060	.000	.015
	P13	.035	.030	.000	.005
	P31	.070	.055	.020	.035
					
UE3 & UE-3	P11	.045	.040	.005	.020
	P13	.030	.035	.000	.010
	P31	.050	.045	.005	.005

P11, P13, and P31, respectively, combine two populations at the ratio of 1 to 1, 1 to 3, and 3 to 1.

### Kangwha study

The Kangwha study was performed to analyze the natural history of BP in Koreans in order to determine important factors associated with changes in BP [13]. In 1986, we initially constructed a cohort of 430 6-year old children who were living in Kangwha Province, Korea. The size of the cohort increased to 715 in 1992 and 784 in 1995. Our case group included blood samples from 101 students(61 boys and 40 girls) who experienced at least once systolic BP measurement of more than 130 mmHg or diastolic BP measurement of more than 85 mmHg between the ages of 15 and 17. Among the 101 samples from the case group, we were also able to obtain blood samples from the parents of 40 subjects and our analysis was based on these 40 probands and their parents.

Angiotensins are substances smaller than proteins that act as vasoconstricting agents, i.e., they cause blood vessels to narrow. Narrowing the diameter of blood vessels increases the blood pressure. ACE, which is located on chromosome 17q23 and has a length of 26 kb, converts angiotensin to its activated functional form, angiotensin-II. There have been several studies investigating the relationship between the ACE gene and high BP [17]. In our analysis we included 4 SNPs, A-240T and C-93T on the promoter, I/D on intron 16, and A2350G on exon 17.

We used the Long, Williams, and Urbanek's method to determine the frequencies of haplotypes comprised of these 4 SNPs and estimated the frequencies of haplotypes, TTDG, ACIA, TTIA, TCIA, ATIA, and ACIG as 41.3, 53.7, 3.1, 0.6, 0.6, and 0.6%, respectively. When estimated by Rohde and Fuerst's method, there were no differences in the frequencies, except that TTDG and ACIA had frequencies of 41.2 and 53.8%, respectively. Table 3 shows the results of our proposed test and Zhao et al's test. The values in the row denoted by 'all SNPs' in the first column of the table represent test statistics and their *p*-values when all 4 SNPs were considered. The *p*-values were obtained by 1,000 randomizations. Neither test showed genetic linkage between ACE and high BP at a significance level of 0.05, but the observed values for each test were quite different.

**Table 3:** Observed values (*p*-value) of proposed test (*T*_*s*_) and Zhao et al's test (*T*_*z*_) for assessing genetic linkage between ACE gene and hypertension

SNPs included	*T*_*s*_	*T*_*z*_
all SNPs	5.275	7.363
(A-240T,C-93T,I/D,A2350G)	(0.232)	(0.152)
		
htSNPs	3.318	4.314
(C-93T,A2350G)	(0.332)	(0.163)

In addition we examined whether any of the 4 SNPs were redundant and if exclusion of those SNPs could affect the outcomes of the two tests. We used a measure of PDE (proportion of diversity explained by a SNP set selection) proposed by Clayton [18] to select so-called htSNPs from the 4 SNPs being considered. This PDE acts like the coefficient of determination in an ordinary regression model. Using the HTSNP procedure [19] on 176 samples of the control group, we identified the htSNP set of C-93T and A2350G with a PDE of 0.992. We repeated the two tests using only the two htSNPs and listed the corresponding results in the row denoted by 'htSNPs' in the first column of Table 3. As was in the case when using all SNPs, using just the htSNPs resulted in no evidence of genetic linkage between ACE and hypertension at a level of 0.05.

## Discussion

Here we propose a score test for linkage between genetic markers and disease-susceptibility genes based on haplotypes. First, we estimated haplotype frequencies using genotypes from affected offspring and their parents. As in [8], we constructed a transmission/non-transmission table of parents' haplotypes to the offspring. Then we proposed a test which mimics Sham's method [14] to test a marginal homogeneity in the transmission/non-transmission table.

Simulations indicate that our test works well at a nominal significant level. Further, we found that the power of the test is monotone and increases as the relative risk increases for dominant models. For recessive models with an unequal haplotype distribution, however, the test was highly conservative due to a low probability of a proband having the mutant homozygotes. In comparison with Zhao et al's test, their test had better power than our test for E case, while our test was more powerful than their test for the UE1, UE2, and UE3 cases. This implies our test has an advantage over Zhao et al's test in the situations where the diversity of haplotypes is low. We also found that our proposed test as well as Zhao et al's test is robust to population admixture. Although both asymptotic chi-squared tests were conservative, Zhao et al's test seemed to be less conservative than our test.

## Conclusion

We propose a score test for linkage between genetic markers and disease-susceptibility genes and show through simulations that our test performed well at a nominal significant level. Our test showed the better performance in power than Zhao et al's test under some situations where the diversity of haplotypes is low. For an application to a Kangwha adolescent cohort, there was no remarkable evidence of genetic linkage between ACE and hypertension.

## Authors' contributions

JK and CN contributed to the formulation of the score test and performed the simulations to investigate its performance. DK applied our test to the Kangwha study data and interpreted the results. All authors read and approved the final manuscript.

## Abbreviations

ACE: Angiotensin-I converting enzyme

BP: Blood pressure

EM: Expectation-Maximization

htSNP: Haplotype tagging single nucleotide polymorphism

RR: Relative risk

TDT: Transmission/disequilibrium test

## Appendix

If the haplotype phases for each parent were identified, the observed set of genotypes is compatible with only one haplotype group. Then t^ij
 MathType@MTEF@5@5@+=feaafiart1ev1aaatCvAUfKttLearuWrP9MDH5MBPbIqV92AaeXatLxBI9gBaebbnrfifHhDYfgasaacH8akY=wiFfYdH8Gipec8Eeeu0xXdbba9frFj0=OqFfea0dXdd9vqai=hGuQ8kuc9pgc9s8qqaq=dirpe0xb9q8qiLsFr0=vr0=vr0dc8meaabaqaciaacaGaaeqabaqabeGadaaakeaacuWG0baDgaqcamaaBaaaleaacqWGPbqAcqWGQbGAaeqaaaaa@3111@ = *t*_*ij *_and thereby Δ^
 MathType@MTEF@5@5@+=feaafiart1ev1aaatCvAUfKttLearuWrP9MDH5MBPbIqV92AaeXatLxBI9gBaebbnrfifHhDYfgasaacH8akY=wiFfYdH8Gipec8Eeeu0xXdbba9frFj0=OqFfea0dXdd9vqai=hGuQ8kuc9pgc9s8qqaq=dirpe0xb9q8qiLsFr0=vr0=vr0dc8meaabaqaciaacaGaaeqabaqabeGadaaakeaaiiqacuWFuoargaqcaaaa@2E27@ = (*t*_1_. - *t*._1_, ..., *t*_*k*-1_. - *t*._*k*-1_)*'*, where *t*_*ij *_is the number of parents with haplotypes *H*_*i*_*H*_*j *_who transmit *H*_*i *_to the affected offspring. Thus the table T^
 MathType@MTEF@5@5@+=feaafiart1ev1aaatCvAUfKttLearuWrP9MDH5MBPbIqV92AaeXatLxBI9gBamXvP5wqSXMqHnxAJn0BKvguHDwzZbqegyvzYrwyUfgarqqtubsr4rNCHbGeaGqiVu0Je9sqqrpepC0xbbL8F4rqqrFfpeea0xe9Lq=Jc9vqaqpepm0xbba9pwe9Q8fs0=yqaqpepae9pg0FirpepeKkFr0xfr=xfr=xb9adbaqaaeGacaGaaiaabeqaamqadiabaaGcbaGafmivaqLbaKaaaaa@37B9@ = {*t*_*ij*_} can be simply considered as a transmission/non-transmission table corresponding to the case of a locus with *k *marker alleles because there are no ambiguities in the parental haplotypes. Let n=2∑g=1Gng
 MathType@MTEF@5@5@+=feaafiart1ev1aaatCvAUfKttLearuWrP9MDH5MBPbIqV92AaeXatLxBI9gBaebbnrfifHhDYfgasaacH8akY=wiFfYdH8Gipec8Eeeu0xXdbba9frFj0=OqFfea0dXdd9vqai=hGuQ8kuc9pgc9s8qqaq=dirpe0xb9q8qiLsFr0=vr0=vr0dc8meaabaqaciaacaGaaeqabaqabeGadaaakeaacqWGUbGBcqGH9aqpcqaIYaGmdaaeWaqaaiabd6gaUnaaBaaaleaacqWGNbWzaeqaaaqaaiabdEgaNjabg2da9iabigdaXaqaaiabdEeahbqdcqGHris5aaaa@394C@. Define *p*_*ij *_as the probability of each parent with haplotypes *H*_*i*_*H*_*j *_transmitting *H*_*i *_to the affected offspring, and the marginal probabilities as pi.=∑j=1kpij
 MathType@MTEF@5@5@+=feaafiart1ev1aaatCvAUfKttLearuWrP9MDH5MBPbIqV92AaeXatLxBI9gBaebbnrfifHhDYfgasaacH8akY=wiFfYdH8Gipec8Eeeu0xXdbba9frFj0=OqFfea0dXdd9vqai=hGuQ8kuc9pgc9s8qqaq=dirpe0xb9q8qiLsFr0=vr0=vr0dc8meaabaqaciaacaGaaeqabaqabeGadaaakeaacqWGWbaCdaWgaaWcbaGaemyAaKgabeaakiabc6caUiabg2da9maaqadabaGaemiCaa3aaSbaaSqaaiabdMgaPjabdQgaQbqabaaabaGaemOAaOMaeyypa0JaeGymaedabaGaem4AaSganiabggHiLdaaaa@3C86@ and p.j=∑i=1kpij
 MathType@MTEF@5@5@+=feaafiart1ev1aaatCvAUfKttLearuWrP9MDH5MBPbIqV92AaeXatLxBI9gBaebbnrfifHhDYfgasaacH8akY=wiFfYdH8Gipec8Eeeu0xXdbba9frFj0=OqFfea0dXdd9vqai=hGuQ8kuc9pgc9s8qqaq=dirpe0xb9q8qiLsFr0=vr0=vr0dc8meaabaqaciaacaGaaeqabaqabeGadaaakeaacqWGWbaCcqGGUaGldaWgaaWcbaGaemOAaOgabeaakiabg2da9maaqadabaGaemiCaa3aaSbaaSqaaiabdMgaPjabdQgaQbqabaaabaGaemyAaKMaeyypa0JaeGymaedabaGaem4AaSganiabggHiLdaaaa@3C86@. Following the arguments of Stuart [20], n[Δ^−E(Δ^)]
 MathType@MTEF@5@5@+=feaafiart1ev1aaatCvAUfKttLearuWrP9MDH5MBPbIqV92AaeXatLxBI9gBaebbnrfifHhDYfgasaacH8akY=wiFfYdH8Gipec8Eeeu0xXdbba9frFj0=OqFfea0dXdd9vqai=hGuQ8kuc9pgc9s8qqaq=dirpe0xb9q8qiLsFr0=vr0=vr0dc8meaabaqaciaacaGaaeqabaqabeGadaaakeaadaGcaaqaaiabd6gaUbWcbeaakiabcUfaBHGabiqb=r5aezaajaGaeyOeI0IaemyrauKaeiikaGIaf8hLdqKbaKaacqGGPaqkcqGGDbqxaaa@3753@ asymptotically follows a (*k *- 1)-variate normal distribution with covariance *n*Σ = {*nσ*_*ij*_}, where *E*(Δ^
 MathType@MTEF@5@5@+=feaafiart1ev1aaatCvAUfKttLearuWrP9MDH5MBPbIqV92AaeXatLxBI9gBaebbnrfifHhDYfgasaacH8akY=wiFfYdH8Gipec8Eeeu0xXdbba9frFj0=OqFfea0dXdd9vqai=hGuQ8kuc9pgc9s8qqaq=dirpe0xb9q8qiLsFr0=vr0=vr0dc8meaabaqaciaacaGaaeqabaqabeGadaaakeaaiiqacuWFuoargaqcaaaa@2E27@) = *E*(*n*(*p*_1_. - *p*._1_), ..., *n*(*p*_*k*-1_. - *p*._*k*-1_))*' *and *σ*_*ii *_= *n*[(*p*_*i*_. + *p*._*i *_- 2*p*_*ii*_) - (*p*_*i*_. - *p*._*i*_)^2^] and for *i *† *j*, *σ*_*ij *_= -*n*[(*p*_*ij *_+ *p*_*ji*_) + (*p*_*i*_. - *p*._*i*_)(*p*_*j*_. - *p*._*j*_)]. From Sham and Curtis [21], under no linkage, *p*_*i*_. = *p*._*i*_, *i *= 1, ..., *k*. Therefore, to test linkage we can use the marginal homogeneity test statistic. Under no linkage, we have *E*(Δ^
 MathType@MTEF@5@5@+=feaafiart1ev1aaatCvAUfKttLearuWrP9MDH5MBPbIqV92AaeXatLxBI9gBaebbnrfifHhDYfgasaacH8akY=wiFfYdH8Gipec8Eeeu0xXdbba9frFj0=OqFfea0dXdd9vqai=hGuQ8kuc9pgc9s8qqaq=dirpe0xb9q8qiLsFr0=vr0=vr0dc8meaabaqaciaacaGaaeqabaqabeGadaaakeaaiiqacuWFuoargaqcaaaa@2E27@) = (0, ..., 0)*' *and, plugging the maximum likelihood estimates, {*t*_*ij*_/*n*}, {*t*_*i*_./*n*}, and {*t*._*j*_/*n*}, of {*p*_*ij*_}, {*p*_*i*_.} and {*p*._*j*_}, respectively into the entries of **Σ**, the estimated covariance matrix *n*Σ^
 MathType@MTEF@5@5@+=feaafiart1ev1aaatCvAUfKttLearuWrP9MDH5MBPbIqV92AaeXatLxBI9gBaebbnrfifHhDYfgasaacH8akY=wiFfYdH8Gipec8Eeeu0xXdbba9frFj0=OqFfea0dXdd9vqai=hGuQ8kuc9pgc9s8qqaq=dirpe0xb9q8qiLsFr0=vr0=vr0dc8meaabaqaciaacaGaaeqabaqabeGadaaakeaaiiqacuWFJoWugaqcaaaa@2E45@ = {*n*σ^ij
 MathType@MTEF@5@5@+=feaafiart1ev1aaatCvAUfKttLearuWrP9MDH5MBPbIqV92AaeXatLxBI9gBaebbnrfifHhDYfgasaacH8akY=wiFfYdH8Gipec8Eeeu0xXdbba9frFj0=OqFfea0dXdd9vqai=hGuQ8kuc9pgc9s8qqaq=dirpe0xb9q8qiLsFr0=vr0=vr0dc8meaabaqaciaacaGaaeqabaqabeGadaaakeaaiiGacuWFdpWCgaqcamaaBaaaleaacqWGPbqAcqWGQbGAaeqaaaaa@316A@}, where σ^ii
 MathType@MTEF@5@5@+=feaafiart1ev1aaatCvAUfKttLearuWrP9MDH5MBPbIqV92AaeXatLxBI9gBaebbnrfifHhDYfgasaacH8akY=wiFfYdH8Gipec8Eeeu0xXdbba9frFj0=OqFfea0dXdd9vqai=hGuQ8kuc9pgc9s8qqaq=dirpe0xb9q8qiLsFr0=vr0=vr0dc8meaabaqaciaacaGaaeqabaqabeGadaaakeaaiiGacuWFdpWCgaqcamaaBaaaleaacqWGPbqAcqWGPbqAaeqaaaaa@3168@ = *t*_*i*_. + *t*._*i *_- 2*t*_*ii *_and for *i *≠ *j*, σ^ij
 MathType@MTEF@5@5@+=feaafiart1ev1aaatCvAUfKttLearuWrP9MDH5MBPbIqV92AaeXatLxBI9gBaebbnrfifHhDYfgasaacH8akY=wiFfYdH8Gipec8Eeeu0xXdbba9frFj0=OqFfea0dXdd9vqai=hGuQ8kuc9pgc9s8qqaq=dirpe0xb9q8qiLsFr0=vr0=vr0dc8meaabaqaciaacaGaaeqabaqabeGadaaakeaaiiGacuWFdpWCgaqcamaaBaaaleaacqWGPbqAcqWGQbGAaeqaaaaa@316A@ = -(*t*_*ij *_+ *t*_*ji*_). Hence *T*_*s *_asymptotically follows a chi-square distribution with (*k *- 1) degrees of freedom.
